# Delivery of Bioactive Gene Particles via Gelatin-Collagen-PEG-Based Electrospun Matrices

**DOI:** 10.3390/ph14070666

**Published:** 2021-07-12

**Authors:** Eleni K. Tsekoura, Teo Dick, Porntipa Pankongadisak, Daniel Graf, Yaman Boluk, Hasan Uludağ

**Affiliations:** 1 Department of Chemical & Materials Engineering, Faculty of Engineering, University of Alberta, Edmonton, AB T6G 1H9, Canada; tsekoura@ualberta.ca (E.K.T.); tadick@ualberta.ca (T.D.); 2 School of Science, Mae Fah Luang University, Thasud, Muang, Chiang Rai 57100, Thailand; pankongadisak.p@gmail.com; 3 Department of Dentistry, Faculty of Medicine & Dentistry, University of Alberta, Edmonton, AB T6G 2R7, Canada; dgraf@ualberta.ca; 4 Department of Civil & Environmental Engineering, Faculty of Engineering, University of Alberta, Edmonton, AB T6G 1H9, Canada; yaman@ualberta.ca; 5 Faculty of Pharmacy & Pharmaceutical Sciences, University of Alberta, Edmonton, AB T6G 2H7, Canada; 6 Department of Biomedical Engineering, Faculty of Medicine & Dentistry, University of Alberta, Edmonton, AB T6G 2V2, Canada

**Keywords:** electrospun matrices, gelatin, collagen, gene delivery, plasmid DNA, Bone Morphogenetic Protein

## Abstract

The fabrication of fiber mats via electrospinning has been adopted in the last decades to produce high quality scaffolds for tissue engineering. However, an effective combination of electrospinning methods with gene delivery therapies remains a challenge. In this study, we describe how the delivery of gene complexes via electrospun mats that contain different volumes of gelatin (Gel), collagen (Col), and polyethylene glycol (PEG) can affect gene expression by transfected cells. Non-viral complexes were formulated by using lipid modified polyethylenimine (PEI) polymer and plasmid DNAs (pDNA) like the reporter Green Fluorescent Protein (GFP) and the therapeutically relevant Bone Morphogenetic Protein-2 (BMP-2) and electrospuned after being mixed with different volumes of Gel-Col-PEG mats and delivered to human myoblast (C2C12) and mouse osteoblast cells (MC3T3). The entrapment of GFP complexes via different homogeneous electrospun fiber mats revealed that a high fraction of collagen in the mats affected the quality of the fibers and led to reduced transfection efficiency on target cells. On the other hand, the fabrication of double-layered mats that contained collagen without complexes as a first layer and gelatin-collagen-PEG with complexes as a second layer successfully induced GFP expression and ALP activity in C2C12 cells. We conclude that this study has established the advantage of formulating multilayered bioactive collagen-based mats for gene delivery applications.

## 1. Introduction

Over the last decade, gene therapy has gained increasing attention in the fields of bone regeneration and tissue engineering [1]. The delivery of gene-based therapeutic agents over protein delivery has been a superior method for the expression or deactivation of a desired protein in target cells or tissues [2,3]. A variety of viral and non-viral vectors has been developed and used for the safe delivery of plasmid DNA (pDNA) based expression systems to the cells. Gene activated matrices (GAMs) have been developed for administration of pDNA in an anatomical area of interest while simultaneously offering a structural support for new tissue and matrix deposition [4,5]. The selection of the most appropriate natural or synthetic scaffold along with the method of processing should be chosen in consideration of the type of interactions between the complexes and the scaffold. The stability of the gene complexes during scaffold fabrication as well as the controlled release rate during scaffold degradation in situ are additional considerations to be addressed [6,7]. A variety of GAM systems, including nanoparticles, hydrogels, freeze dried scaffolds, and electrospun membranes have been investigated with the aim of the successful delivery of complexes to cells of interest [8,9,10,11]. Most GAMs are fabricated by preparing the scaffolds and gene complexes separately and formulating them together before administration in a biological system. Electrospun fibers have become increasingly attractive as a scaffold due to their structural similarities to that of extracellular matrix (ECM) [12]. The electrospinning process involves several important parameters, including polymer properties such as MW, solution properties such as viscosity, process parameters such as applied voltage, flow rate and drying time, and environmental conditions such as temperature and humidity. All these parameters can have an impact on the final nanofiber properties and the release/presentation profile of the genes [13,14].

Gelatin has been successfully used as a natural polymer for scaffolds fabrication due to its biocompatible and biodegradable properties [15]. As a natural polymer, gelatin is water-soluble but for the formation of electrospun fibers, organic solvents such as 2,2,2-Trifluoroethanol (TFE) and 1,1,1,3,3,3-Hexafluoro-2-propanol (HFP) are usually needed [16,17]. Gelatin has been previously investigated as a pDNA delivery carrier; Pankongadisak et al. proposed Gelatin-Polyethyleneglycol (PEG) electrospun fibers for the delivery of pDNA complexes via mats. The study showed a robust Alkaline Phosphatase (ALP) induction by delivering gene complexes based on a pDNA expressing Bone Morphogenetic Protein-2 (pBMP-2) to C2C12 and MC-3T3 cells via Gelatin-PEG fibers [18]. However, the gelatin’s rapid dissolution under physiological conditions makes it undesirable for long-term delivery of pDNA that is typically needed for bone tissue engineering. The degradation (or resiliency) of gelatin can be altered by changing the gelatin source, its MW, the degree of crosslinking or by adding another synthetic/natural polymer to the gelatin formulation depending on the final application [19,20].

In this work, we aimed to fabricate new electrospun scaffolds as GAM by incorporating collagen Type I into the mats. Collagen Type I, as the main structural component of the extracellular matrix (ECM), has been considered a more physiological option for scaffolds that are intended to be used in tissue engineering. To create such functional scaffolds, this study initially explored the formation of homogenous Gelatin-Collagen-PEG mats that contained different volumes of collagen in addition to pDNA complexes, as in Pankongadisak et al. [18]. In this case, we observed a detrimental effect on the delivery of complexes into the cells after the incorporation of collagen into the mats. Hence, we explored the formation of two-layered mats that contained collagen fibers without complexes as the first layer and as a second layer a mixture of Gelatin, Collagen, PEG and pDNA complexes. The pDNA complexes were created using a commercially available lipid-modified polyethylenimine (PEI) transfection reagent. The mats bearing gene complexes were evaluated for transgene expression and induction of osteogenic activity in vitro by using the well-established C2C12 and MC3T3 cell models. We showed that the two-layered mat formulation is more promising as GAMs for tissue engineering applications

## 2. Results

### 2.1. SEM Imaging of Gene-Activated Mats

**Figure 1:**
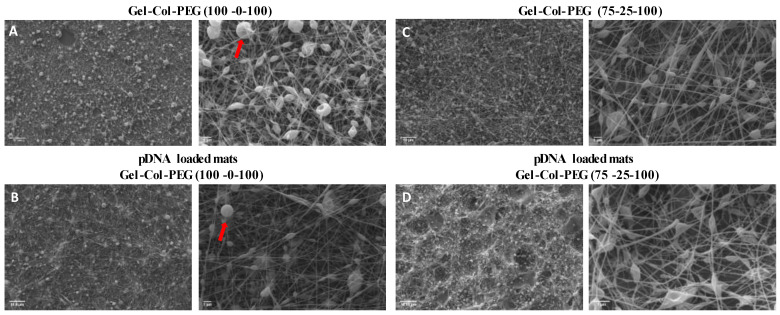

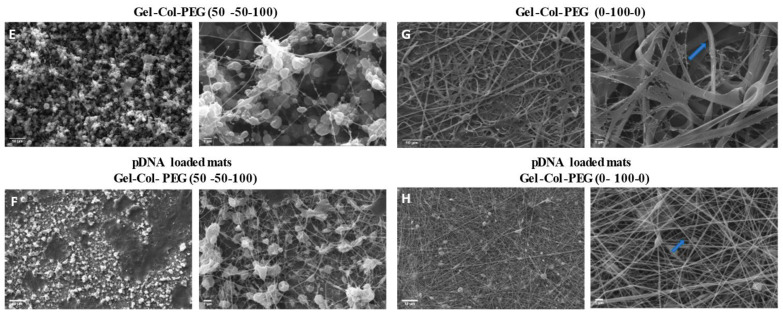
SEM images of different volume ratio Gelatin-Collagen-PEG electrospun fibers without and with complexes; Gelatine-Collagen-PEG (100-0-100) fiber mat without (**A**) and with pDNA complexes (**B**); Gelatine-Collagen-PEG (75-25-100) fiber mat without (**C**) and with pDNA complexes (**D**); Gelatine-Collagen-PEG (50-0-50) fiber mat without (**E**) and with pDNA complexes (**F**); Gelatine-Collagen-PEG (0-100-0) fiber mat without (**G**) and with pDNA complexes (**H**) (scale bars 10 and 1 μm). Red arrows show some beads along the nanofibers (blue arrows).

The morphology and average diameter of the electrospun fibers are shown in Figure 1 and Table 1, respectively. Four different volume ratio Gelatin-Collagen-PEG mats with and without complexes were prepared and characterized through SEM observations. As shown in Figure 1A,B, the Gelatin-Collagen-PEG (100-0-100) mat with and without complexes showed poor quality of fibers containing a large number of beads. The average fiber diameter without complexes was 90 ± 20 nm and the bead size was 663 ± 215 nm. For the electrospun fibers carrying the pDNA complexes, the fibers diameter increased to 97 ± 6 nm and the bead size to 770 ± 111 nm. Similar quality of fibers was observed from the Gelatin-Collagen-PEG (75-25-100) mats (Figure 1C,D); the presence of collagen in the electrospun mixture increased the fiber diameter to 140 ± 10 nm and bead’s size to 706 ± 95 nm of the mats without complexes, while the fiber diameter decreased to 126 ± 32 nm and the bead size to 330 ± 27 nm in the presence of complexes. By increasing further, the collagen content in the mats as in the case of Gelatin-Collagen-PEG (50-50-100) (Figure 1E,F), the quality of fibers was even lower given by the higher number and sizes of beads in the mats. This was the case for both with and without pDNA complexes electrospun mats. In contrast, uniform fibers without the presence of beads and an average diameter of 146 ± 38 nm were observed by electrospinning just collagen (Gelatin-Collagen-PEG (0-100-0) mats) without pDNA complexes as illustrated in Figure 1G. In the presence of pDNA complexes (Figure 1H), good quality fibers were also obtained, but the fiber size was increased to 177 ± 31 nm and the size of the observed beads was 770 ± 99 nm.

### 2.2. Delivery of Cy3-pDNA-Loaded Complexes via Mats

**Figure 2:**
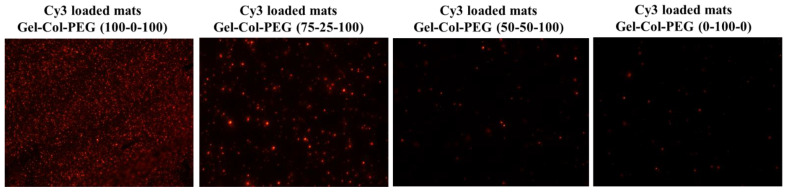
Fluorescence microscopy images of different volume ratio of Gelatin-Collagen-PEG mats with Cy3-labeled pDNA complexes.

To evaluate the presence and distribution of pDNA complexes in the fiber mats, the electrospun monolayer mats were prepared by using Cy3-labeled pDNA complexes, whose presence and distribution in the prepared mats were examined by fluorescence microscopy. As shown in Figure 2, the presence of Cy3-complexes in collagen-free Gelatin-Collagen-PEG (100-0-100) mats showed the strongest entrapment and most-uniform red (Cy3) signal, which confirmed the successful entrapment of complexes in the mat. The presence of the red Cy3-complexes appeared to be less in the next set of mats, as the collagen content in the mats was increasing and gelatin content was decreasing. With 25% collagen, a punctuate pattern of Cy3-pDNA complexes were evident, indicating some aggregation of the complexes.

### 2.3. Particle Size and Charge Analysis

The pDNA complexes released from the monolayered fiber mats were analyzed for hydrodynamic size (Z-average) and surface charge/ζ-potential (Table 2). The complexes were prepared by mixing pDNA with ALL-Fect transfection reagent, where polyaspartic acid (pAsp) was added to pDNA solution in some cases. The mean size of the pDNA complexes without the addition of pASP at ALL-Fect:pDNA ratios of 10:1 was 101.3 nm, while the mean size of the formulation with the additive (pDNA/pASP ratio of 1) was 90.0 nm. The mean size of the ALL-Fect/pDNA/pASP complexes released from Gelatin-Collagen-PEG (100-0-100) mat was 113.3 nm and it was further decreased to 42.0 nm in the case of complexes entrapped in the Gelatin-Collagen-PEG (0-100-0) mat. Overall, the released particles that have been incorporated into the mats with high collagen volume ratio were smaller in size than the particles that have been entrapped in the mats without collagen Figure S1 in Supporting Information shows the correlation function and size distribution of dissolved mats with and without complexes. The ζ-potential values were decreased slightly from 38.0 to 35.5 mV after the incorporation of pASP in the complexes. After the encapsulation of the complexes in Gelatin-Collagen-PEG (100-0-100) and (75-25-100) mats, the released particles showed ζ-potential values that were half the value of the original particles. In the absence of added pDNA complexes, no particles were detected in this analysis, indicating that the mats incubated in the aqueous buffers did not release any particles into the solution.

### 2.4. Transfection Efficiency

**Figure 3:**
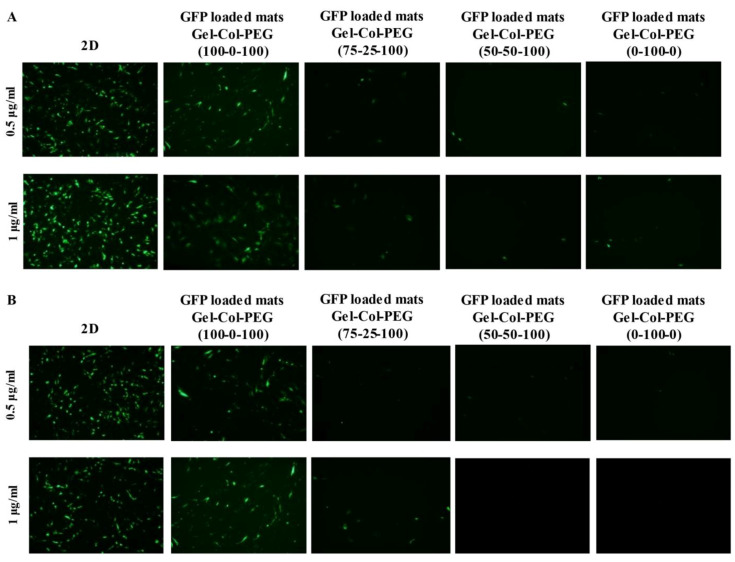
Delivery of gWIZ-GFP complexes via different volume ratio Gelatin-Collagen-PEG mats to C2C12 (**A**) and MC-3T3 (**B**) cells. GFP expression was qualitatively assessed 48 h after transfection using fluorescent microscopy. The percentage of gelatin, collagen and PEG in monolayer mats used is indicated on top of the micrographs where on the left are the pDNA concentrations (1 and 0.5 μg/mL) in cell culture medium carried by the complexes.

**Figure 4:**
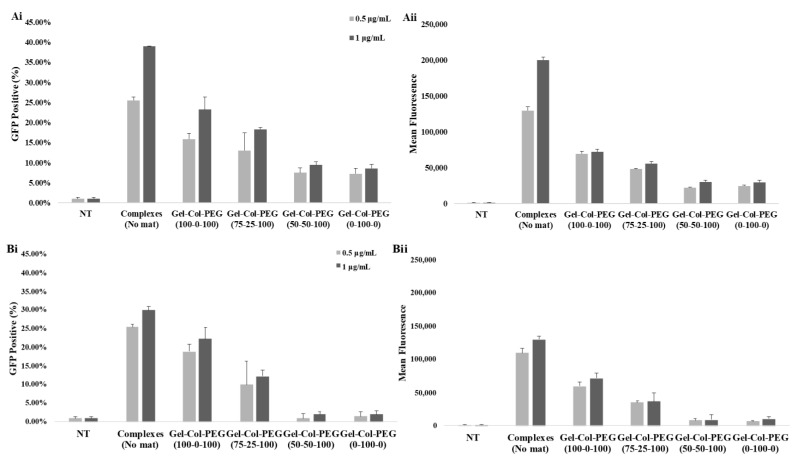
In vitro transfection in C2C12 (**Ai**,**Aii**) and MC3T3 (**Bi**,**Bii**) cells analyzed 48 h post-transfection by flow cytometry. The different volume Gelatin-Collagen-PEG mats were loaded with GFP complexes. The GFP concentrations in cell culture medium were 1.0 and 0.5 μg/mL. NT: no treatment.

The delivery of pDNA complexes to C2C12 and MC-3T3 cells from different monolayer mats was evaluated by fluorescent microscopy (Figure 3A,B) and flowcytometer (Figure 4A,B) 48 h post-transfection. Two concentrations of the pDNA complexes were examined and the encapsulation efficiency from the mats was assumed at 100%. Transfection by free (un-electrospun) complexes on cells on tissue culture plastic served as the positive reference treatment, which indicated strong transfection in both cell types (Figure 3A,B—left micrographs). In both cell lines, the presence of collagen in the mats showed a negative effect on the transfection efficiency, where the transfection efficiency was decreased as the collagen percentage in the mats increased. In addition, a significant difference in transfection efficiency was observed between the delivery of free pDNA complexes and the complexes encapsulated into the different monolayer mats.

For the quantitative analysis of the GFP expression levels in C2C12 (Figure 4Ai,Aii) and MC-3T3 (Figure 4Bi,Bii) cells, the complexes with pASP were encapsulated in different Gelatin-Collagen-PEG mats and then the GFP expression levels in the cells were analyzed using flow cytometer. The mean fluorescence intensity and the percentage of GFP expression were plotted against the different volume ratio Gelatin-Collagen-PEG mats. In C2C12 cells, based on the GFP positive cell population (Figure 4Ai), an increase on GFP expression was observed after increasing the pDNA concentration from 0.5 to 1.0 μg/mL; 25% to 40%, 16% to 23%, and 13% to 18% for free complexes (no mat), 100-0-100 Gelatin-Collagen-PEG (100-0-100) and Gelatin-Collagen-PEG (75-25-100), respectively, while for Gelatin-Collagen-PEG (50-50-100) and (0-100-0) mats, GFP expression remained at ~5% for both pDNA concentrations. The mean fluorescence associated with cells (Figure 4Aii) also showed a similar pattern for the free complexes and complexes delivered within different mats.

The data from the delivery of complexes to MC-3T3 cells are summarized in Figure 4Bi,Bii. In general, the incorporation of complexes in the different volume ratio mats reduced the percentage of GFP expression and mean fluorescence compared to the free complexes independent of the examined pDNA concentration. The delivery of free complexes (no mat) increased the GFP expression from 26% to 30% following the increase of pDNA concentration from 0.5 to 1.0 μg/mL, while for complexes delivered via mats, the GFP expression increased from 19% to 22% for Gelatin-Collagen-PEG (100-0-100) and from 10% to 12% for Gelatin-Collagen-PEG (75-25-100) mats, respectively. Finally, in the cases of Gelatin-Collagen-PEG (50-50-100) and (0-100-0) mats, the presence of higher collagen content significantly decrease the mean GFP values with only 3% of the cell population to display GFP fluorescence.

### 2.5. Double-Layer vs. Monolayer Mats

**Figure 5:**
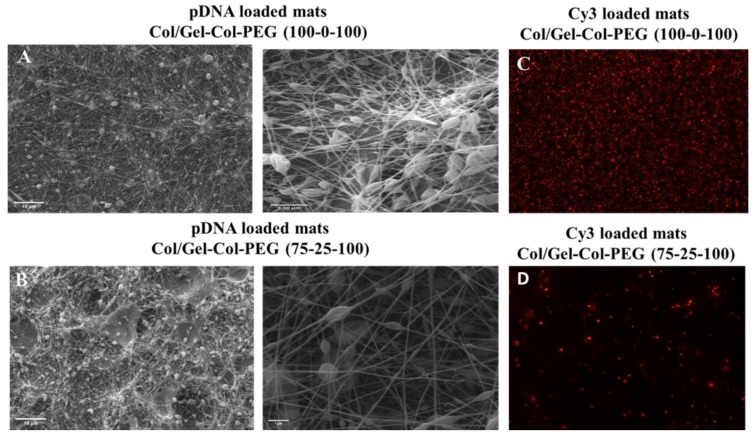
The SEM images (**A**,**B**) of multilayered Collagen/Gelatin-Collagen-PEG (**A**): (100-0-100) and (**B**): (75-25-100)) mats with complexes (scale bars 10 and 1 μm). Fluorescence microscopy images of multilayered Collagen/Gelatin-Collagen-PEG mats (**C**): 100-0-100 and (**D**): 75-25-100) mats with Cy3-labeled pDNA complexes.

Given the detrimental effect of collagen on the bioactivity of electrospun complexes, we decided to prepare double-layered mats by first electrospinning pure collagen solution (i.e., Gelatin-Collagen-PEG of (0-100-0)) and then Gelatin-Collagen-PEG (100-0-100) or Gelatin-Collagen-PEG (75-25-100) fibers to produce double layer mats composed of Collagen/Gelatin-Collagen-PEG (100-0-100) and Collagen/Gelatin-Collagen-PEG (75-25-100). The morphology of the double-layered electrospun fibers was characterized by SEM (Figure 5A,B). Both mats with pDNA complexes showed fibers containing a large number of beads similar to the monolayered mats (Figure 1A–D). The Collagen/Gelatin-Collagen-PEG (100-0-100) and Collagen/Gelatin-Collagen-PEG (75-25-100) double-layer mats had fibers diameter of 98.2 ± 3 nm and 138.6 ± 6 nm and beat size of 769 ± 85 nm and 705 ± 110 nm, respectively. The presence of pDNA complexes in mats was evaluated by using Cy3-labeled pDNA. Similar to the monolayer mats (Figure 2), the double-layered Collagen/GelatinCollagen-PEG (100-0-100) mats showed the strongest red signal (Figure 5C) while the Collagen/Gelatin-Collagen-PEG (75-25-100) displayed lower number of complexes (Figure 5D).

**Figure 6:**
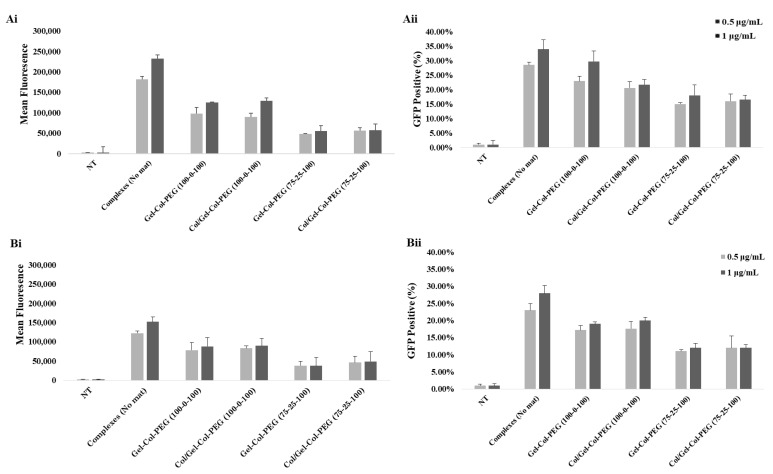
GFP expression by C2C12 (**Ai**,**Aii**) and MC3T3 (**Bi**,**Bii**) cells treated with monolayer mats Gelatin-Collagen-PEG (100-0-100) and (75-25-100) and double layered Collagen/Gelatin-Collagen-PEG (100-0-100) and (75-25-100) mats containing GFP complexes. The transfection efficiency was summarized as GFP-positive population (**Ai**,**Bi**) and as mean fluorescence intensity/cell (**Aii**,**Bii**) 48 h post-transfection by flow cytometry analysis. The GFP concentrations in cell culture medium were 1.0 and 0.5 μg/mL. NT: no treatment.

The transfection efficiency of electrospun pGFP complexes in C2C12 (Figure 6Ai,Aii) and MC-3T3 (Figure 6Bi,Bii) cells was also quantitatively evaluated by flow cytometer. The mean fluorescence intensity and the percentage of GFP expression were plotted against the different volume ratio Collagen/Gelatin-Collagen-PEG and Gelatin-Collagen-PEG mats. In C2C12 cells, based on the mean fluorescence and GFP positive cell population results (Figure 6Ai), an increase in transfection was observed after increasing the pDNA concentration from 0.5 to 1.0 μg/mL for free complexes and complexes delivered by Collagen/Gelatin-Collagen-PEG (100-0-100). No difference in the mean fluorescence and GFP results was observed between the two examined concentrations for Collagen/Gelatin-Collagen-PEG (75-25-100). In addition, similar values were recorded between monolayer and double-layered mats, where the mats having collagen electrospun with complexes (i.e., Gelatin-Collagen-PEG of (75-25-100)) led to lower transfection efficiency of the complexes in the cells. Similar results to C1C12 cells were also highlighted by the MC-3T3 cells. Taken together, the delivery of complexes to the cells was equivalent between the single-layer and double-layer format, and the presence of the first layer of collagen did not significantly affect the delivery of complexes to the cells.

### 2.6. Induction of ALP Activity by Complexes from Double vs. Monolayer Mats

**Figure 7:**
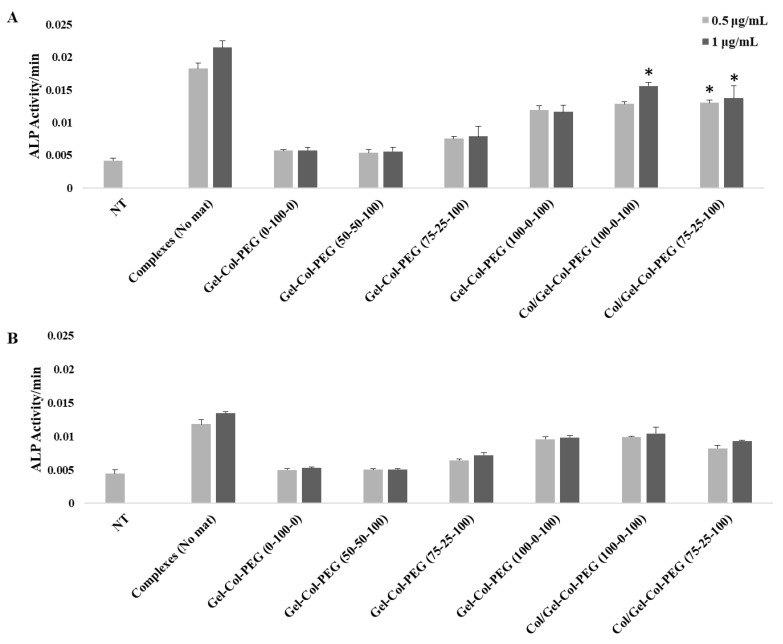
ALP induction in C2C12 cells (**A**) and in MC3T3-E1 (**B**) cells after delivery of gWIZ-BMP-2 complexes from different Gel-Col-PEG monolayer mats and double-layered mats 7 days post transfection. The pDNA concentrations in cell culture were 0.5 and 1.0 μg/mL. The ALP activity was measured 30 min after the addition of the substrate in triplicate. * *p* < 0.05 compared with monolayer mats.

The delivery of BMP-2 expression plasmid to the cells was evaluated by quantifying the induced ALP activity in transfected cells. The complexes were incorporated into four different Gelatin-Collagen-PEG mats and incubated with the cells over seven days. The estimated pBMP-2 concentrations that the cells were exposed were 1.0 and 0.5 μg/mL, but the ALP induction results were equivalent at both concentrations. As shown in Figure 7A,B, the delivery of free complexes increased the ALP levels in both cell lines, as expected. In both cell types, the cells that were treated with complexes released from the Gelatin-Collagen-PEG (0-100-0) and (50-50-100) mats did not affect the ALP induction (i.e., ALP levels similar to the untreated cells). On the other hand, the cells that have been treated with complexes delivered via mats without collagen Gelatin-Collagen-PEG (100-0-100) showed a relatively high ALP activity compared to the other examined mats. As it was highlighted above, the presence of Collagen in the monolayered mats decreased the ALP activity of the cells. Lastly, the C2C12 cells that have been treated with Collagen/Gelatin-Collagen-PEG (100-0-100) and Collagen/Gelatin-Collagen-PEG (75-25-100) double-layered scaffolds displayed a significantly increase ALP activity (*p* < 0.05) compared to the monolayer mats. In the case of MC-3T3 cells, there was no significant difference in the ALP activity values between the multilayered and monolayered mats.

## 3. Discussion

The evolution of gene activated matrices in medicine have opened a new era in the effective delivery of expression vectors via non-viral carriers. Producing nanofibers via electrospinning technique is considered a suitable method for the delivery of nanoparticles even though problems associated with interactions between the nanoparticles and the scaffold are still unsolved [21]. To overcome these limitations, the selection of the right fabrication method as well as the most suitable biomaterial for the application is critical. Electrospinning as a fabrication method has attracted significant attention since good control over the solution properties of the starting materials, process parameters, and environmental conditions is possible to fine-tune the final nanofiber properties and the release profile of any entrapped bioactive agents [3,14]. Gelatin derives from controlled hydrolysis of fibrous insoluble collagen and has been widely used to fabricate hydrogels, fibers, and microspheres for numerous biomedical applications. However, its poor mechanical properties and low thermal stability is limiting further applications of gelatin [22]. Therefore, the modification of gelatin including blending with other natural or synthetic polymers has been explored [23,24]. In our previous study, the incorporation of PEG as a modifier into gelatin solution significantly improved the spinnability of the gelatin and enabled successful delivery of pDNA complexes to C2C12 and MC-3T3 cells [18]. In the present work, in order to further improve the limiting features of gelatin/PEG composite fibers, collagen Type I was incorporated into spinning process with two different ways; (i) by incorporating collagen in the gelatin-PEG solution to create homogenous (monolayer) mats or (ii) by spinning first collagen solution as the first layer of the mat, followed by the deposition of a second layer of Gelatin-Collagen-PEG mat as a double-layered scaffold. Collagen type I, as the main ECM protein of many tissues, should closely mimic the native ECM tissue and is highly desirable for improved tissue regeneration [25]. The pDNA complexes are likely to be released as a result of gelatin dissolution under culture conditions (the setting of the current study) and their features should allow uptake in cells in the vicinity of mats.

When the morphology of the fibers was examined using SEM, a large number of beads was intertwined in all the mats that contained Gel and PEG, which indicates the poor electrospinnability of the solutions. With an increase in the collagen content, the quality and the diameter of the composite nanofibers changed, and a dramatically larger number of beads was found. This was most likely due to the decrease in the solution’s viscosities and thus an increased number of beads occurred [26]. In contrast, the electrospinning of pure collagen without complexes created uniform fibers with average diameter of 146 nm without beads, demonstrating high electrospinnability of pure collagen solution. The good quality of collagen fibers was also confirmed after the encapsulation of pDNA complexes, where the diameter of fibers increased to 177 nm with the appearance of a small number of beads. The beads are presumably due to the presence of nanoparticle size complexes in the spinning solution, which may have also increased the fiber diameters. Our studies showed once more that electrospinning can produce uniform and good quality collagen fibers with diameter between 50 to 1200 nm as has been reported in the literature by others [27].

Delivering pDNA complexes to the cells by encapsulating them in electrospun fibers may affect the physicochemical properties of the complexes as well as may affect the transfection efficiency [28]. It is expected that this mode of delivery will be superior for gene activated matrices where the therapeutic genes are usually incorporated into the matrix after the matrix is fabricated [29,30]. The influence of blending different volumes of Gel, Col, and PEG with complexes had an impact on the NPs properties. Notably, the size of the complexes released from the Gelatin-Collagen-PEG (100-0-100) and (75-25-100) mats were similar to the free complexes, but the ζ-potential appeared to be lower. Both the size and ζ-potential dropped further as the volume of collagen increased and Gel decreased, showing the negative effect of adding high amount of collagen into the electrospun solution. It is likely that the collagen was involved in the coating of the complexes and reduced the ζ-potential, but this remains to be experimentally verified. Electrospinning Cy3-labeled pDNA complexes into the mats confirmed the successful entrapment of complexes, but in line with the size measurements, less abundant and smaller fluorescent particles were evident especially after increasing the collagen content in Gelatin-Collagen-PEG mats. In subsequent experiments, we confirmed the negative effect of delivering complexes via mats with large collagen content on the transfection efficiency and ALP activity against both cell lines.

In order to ascertain the influence of collagen on the transfection efficiency, we examined the delivery of gWIZ-GFP complexes from monolayer (homogeneous) mats to C2C12 and MC-3T3 cells. The experimental procedure that was used here involved extraction of the complexes with trypsin and exposure of the cells to the complexes. This was considered necessary, since unlike Gel-PEG mats, mats with collagen did not dissolve right away in tissue culture medium, which would have complicated assessment of transfection efficiency due to possible slower release profile of complexes. Initial studies indicated that incubation of complexes (free) in the employed trypsin concentration/time did not affect the transfection efficiency of the complexes (Figure S2). The data from our experimental system revealed that the transfection efficiency was higher with Gelatin-Collagen-PEG (100-0-100) and (75-25-100) mats than with those containing higher volume of collagen, suggesting that the presence of collagen in the fibers did not enhance transfection. A small amount of collagen appeared to be tolerated but not higher amounts. The reduced transfection efficiency was evident in both cell lines, and we attributed this to (i) decrease in the amount of pDNA entrapped and released from the mats (based on entrapment efficiency of visualized Cy3-labeled pDNA), and (ii) smaller and less charged complexes with reduced sedimentation and possibly binding to anionic cell surfaces. Similar results have been observed in other controlled release studies, suggesting that a high matrix density can entrap bioactive molecules. In the case of Saraf et al., the delivery of pDNA/PEI-HA complexes in coaxial PCL/PEG electrospun matrices was dependent on the PEG concentrations, higher PEG concentrations were leading to a decrease of the transfection efficiency due to the lower amount of pDNA released from the fibers [31]. We observed a significant effect on the transfection efficiency between the free and entrapped complexes. This is not unexpected given the possible adverse effects from the process of entrapment; in our previous studies with Gelatin-based electrospun matrices, we typically recovered 50% of the complexes in the electrospun matrices, whereas in the current study we assumed 100% recovery when small amount of collagen was present during electrospinning. The relatively small reduction in the obtained transfection efficiencies from the free complexes provides encouragement to further optimize the transfection efficiency of gene-activated electrospun mats.

The BMPs and in particular BMP-2 play an important role during osteogenesis since they can promote osteogenesis by up-regulating ALP, and other mediators, during the process of calcification [32]. For this study, the induction of osteogenic activity was measured by delivering BMP-2 expressing pDNA to C2C12 and MC-3T3 cells. The delivery of BMP-2 protein to C2C12 cells has the ability to convert their myoblast phenotype into cells displaying osteoblastic features [33]. On the other hand, MC-3T3 cells is a preosteoblasts cell line with the capacity to enhance the differentiation into osteoblast and osteocytes upon the right stimulation. The delivery of BMP-2 complexes significantly induced the ALP activity in both cell lines after seven days of incubation, further confirming the bioactivity of the entrapped complexes as observed with gWIZ-GFP plasmids. As in later complexes, results with the BMP-2 plasmid transfection also indicated a detrimental role of excess collagen incorporation in the Gelatin-Collagen-PEG mats.

To create more effective electrospun mats, we employed an alternative strategy to produce a dual-layered mat, where different polymeric solutions were spun individually to form a two-layered structure [34]. The multilayered scaffold proposed here was intended to address the main challenge of monolayered mats, where the high volume of collagen prevented effective transfection. Based on the results from monolayered mats, we decided to first fabricate a collagen layer without complexes and then overlay a second layer of Gelatin-Collagen-PEG (100-0-100) or (75-25-100) bearing the complexes. We observed no differences between the monolayered and double-layered mats from the SEM as well as the images of Cy3-labeled pDNA complexes. Similarly, no detrimental effects were observed from the delivery of GFP complexes from double-layered mats to C2C12 and MC-3T3 cells. We also demonstrated a significant ALP activity in C2C12 cells after the delivery of complexes from double-layered mats. The ALP induction was higher in comparison to monolayer counterparts as long as the collagen was electrospun separately from the complexes. This presumably reduces undesirable interactions between the collagen and the complexes in the mats. On the other hand, the difference in the ALP levels for BMP-2 complex treated cells were not as high as in the MC-3T3 cells after one week of incubation. It is possible that the one week of incubation time for MC-3T3 cells might not have allowed for the differences to be revealed. A more systematic study is warranted in this regard. Nevertheless, we can still see an induction of ALP activity in MC-3T3 cells for the most optimal mat, Gelatin-Collagen-PEG of (100-0-100) (Figure 7B; i.e., twice the background). Having a Collagen layer in addition to the mats fabricated with Gelatin-Collagen-PEG of (100-0-100) and pBMP-2 complexes did not significantly support the osteogenic activity of the complexes. Clearly separating the Collagen from complexes during electrospinning helped to preserve the activity of them.

## 4. Materials and Methods

### 4.1. Materials

The fetal bovine serum (FBS), ALP substrate p-nitrophenol phosphate (p-NPP), gelatin Type A (300 Bloom; porcine skin), and 2,2,2-Trifluoroethanol (TFE) (N99.0%) were purchased from Sigma-Aldrich (St Louis, MO, USA). The PEG (Mn = 20 kDa) was obtained from Fluka (Buchs, Switzerland). The polyaspartic acid (pASP) was purchased from Alamanda Polymers (Huntsville, AL, USA). Dulbecco’s Modified Eagles Medium (DMEM)/F12 medium (1:1) (1×, with L-glutamine and 15 mM HEPES), Hank’s Balanced Salt Solution (HBSS), penicillin/streptomycin (10,000 U/mL/10,000 μg/mL), GlutaMax-I (100×), and MEM NEAA (100×) were from Gibco (New York, NY, USA). All-Fect transfection reagent was obtained from RJH Biosciences Inc. (Edmonton, AB, Canada). The gWIZ-GFP and gWIZ plasmids were purchased from Aldevron (Fargo, ND, USA). Cy3 used for pDNA labeling was from Mirus (Madison, WI, USA)

Type I collagen was extracted from rat tail tendons via acid dissolution. Prior to use, the rat tails were disinfected with 70% ethanol by submerging and storing at −20 °C. The tendons were removed from the rat tails and washed with Tris buffered saline (0.9% NaCl, 10 mM Tris). They have been weighted and dehydrated with a serial concentration of ethanol (50%, 75%, 95%, and 100%, ~30 min each). Afterwards, they were added into pre-cooled 0.5 M acetic acid (100 mL/g wet tendon) and stirred at 4 °C for 48–72 h. After the incubation time with acetic acid, the tendons solution were centrifuged and supernatant was added to an equal volume of pre-cooled 10% NaCl for precipitation overnight at 4 °C. The next day, the floated collagen was harvested and centrifuged. The collagen pellets were collected and resuspended in 0.25 M acetic acid at 4 °C (100 mL/g initial tendons). Later, the collagen-acetic acid solution was dialyzed against 0.025 M acetic acid at 4 °C for three days and the buffer was changed twice a day. For the last two changes, the buffer was replaced with ddH_2_O. Finally, the resulting dialyzed collagen solution was freeze-dried for 48 h and the obtained powder was stored at 4 °C prior to any further use.

### 4.2. Preparation of Complexes

The complexes were prepared at room temperature by adding first a desired volume of ALL-Fect transfection reagent (1 mg/mL) to serum-free medium (DMEM/F:12) and then an aqueous solution that contained pASP (0.4 μg/μL) and GFP-pDNA (0.4 μg/μL). The final ratio of ALL-Fect:pDNA was 10:1, and the final ratio of pDNA:pASP was 1:1. Complexes were incubated for 30 min at room temperature prior to use in electrospinning or direct transfection of the cells.

### 4.3. Fabrication of Gene-Activated Electrospun Mats

#### 4.3.1. Monolayer Mats

The gene-activated mats were prepared using a similar approach as Pankongadisak et al. [18]. Briefly, gelatin (100 mg/mL) and collagen (50 mg/mL) were dissolved in TFE solution and PEG (10 mg/mL) was dissolved in dH2O. For the fabrication of the mats, different volume ratio Gel-Col-PEG mixtures were prepared at 100-0-100, 75-25-100, 50-50-100, and 0-100-0. For the mats loaded with complexes, the complexes were prepared prior to electrospinning as described in Section 4.2 and dispersed in the different volume ratio Gel-Col-PEG solutions at a volume ratio of 1:3, respectively, and mixed well before electrospinning. The final solution was transferred into a 1-mL plastic syringe with a 20-gauge needle. The mats were electrospun onto an aluminium foil by using an AL-4000 programmable syringe-pump. The conditions for mat production were (i) flow rate of 350 μL/h, (ii) applied voltage 19 kV, and (iii) working distance to foil of 10 cm. The final mats were collected and dried under a biological safety cabinet for 30 min and disinfected under UV light for 10 min prior to testing.

#### 4.3.2. Double-Layered Mats

For the first layer, 50 μg/mL collagen Type I in TFE was electrospun first by using the same electrospinning parameters described in Section 4.3.1. For the second layer, a solution containing Gel-Col-PEG (100-0-100 or 75-25-100) with complexes at a volume ratio 1:3, respectively, was electrospun onto the collagen mat by using the same parameters. The final mats were collected and dried under a biological safety cabinet for 30 min and disinfected under UV light for 15 min prior to any further use.

### 4.4. Characterization of Gene-Activated Electrospun Mats

The morphology of the electrospun mats with and without complexes was observed by SEM operated at an acceleration voltage of 10 kV. Before observation, the mats were cut into 1 cm × 1 cm pieces and coated with gold to increase conductivity. Fiber diameters were measured by using the Image J 1.52 V software. The presence of complexes in mats was determined by preparing the mats as described in Section 4.3 except that the complexes were carrying Cy3-labeled pDNA as described in Pankongadisak et al. [18]. The mats were collected and then observed under the Olympus FSX100 Fluorescence Microscope. The particle size and surface charge of the complexes used for electrospinning was determined by using a Litesizer 500 system (Anton Paar, Austria) with dynamic light scattering and zeta potential measurements, respectively. The mats with and without complexes were dissolved with 0.05% trypsin and incubated in a water bath for 5 min at 37 °C. Next, equal amount of DMEM/F12 was added and finally the solution was diluted with free RNA water. Freshly free complexes were also prepared as controls at room temperature. Prior to measurements all the samples have been filtered.

### 4.5. Cell Culture

Mouse myoblast C2C12 cells and cloned mouse calvarial osteoblast MC3TC-E1 cells were used as model cell lines. C2C12 cells were maintained in DMEM/F:12 (1:1) supplemented with 10% FBS, 100 U/mL penicillin, 100 μg/mL streptomycin, 0.1% GlutaMax-I and 0.1% NEAA. The MC3TC-E1 were maintained in 50% DMEM/F:12 (1:1) and 50% AMEM supplemented with 10% FBS, 100 U/mL penicillin, 100 μg/mL streptomycin, 0.1% GlutaMax-I and 0.1% NEAA. The cells were routinely maintained under humidified atmosphere (95/5% air/CO_2_) at 37 °C in the indicated cell culture medium.

### 4.6. Transfection Efficiency of Gene Activated Mats

Twenty-four hours prior to transfection experiments, the cells were seeded in 48-well cell culture plates at a density of 10^4^ cells/cm^2^. The mats fabricated as described in Section 4.3.1 carrying the gWIZ-GFP complexes were first incubated in 200 μL of 0.05% trypsin and incubated for 5 min at 37 °C water bath. Then, 200 μL of DMEM/F12 were added to stop the reaction. A predetermined volume of the final solution was added to the cells to give final pDNA concentrations of 0.5 and 1 μg/mL in tissue culture medium. Free complexes were also prepared and mixed with trypsin and DMEM/F12 solution (as above) prior to adding them into the cells. Free complexes without any treatment were also prepared and added directly to the cells to serve as a positive control treatment. The transfection efficacy was investigated 48 h post-transfection by Olympus FSX100 Fluorescence Microscope and by flow cytometer. For flow cytometer analysis, the cells were washed (×3 times) with HBSS, trypsinized with 0.05% trypsin, and fixed with 3.7% formalin in HBSS. The transfection efficiency was quantified based on GFP positive population and the mean fluorescence intensity of the cells by BD LSRFortessa (Becton-Dickinson, San Jose, CA, USA). Each study group contained three replicates.

### 4.7. Delivery of BMP-2 Plasmid from Gene Activated Mats

The cells were seeded in 24-well plates the day before the experiments. Gene activated mats loaded with BMP-2 plasmid were prepared as described in Section 4.3.1 and Section 4.3.2 and added to the cells as was explained in Section 4.6. One-week post-transfection, the cells were washed with HBSS (×2 times) and lysed with 200 μL ALP buffer (0.5 M 2-amino-2-methylpropan-1-ol and 0.1% Triton-X; pH:10.5) for 2 h at room temperature under constant shaking. A total of 200 μL of lysed cell solution from each well was incubated with 200 μL of 2 mg/mL ALP substrate (p-NPP) for 30 min. Afterwards, the ALP activity was determined by measuring the absorbance at 405 nm by using the ELx800 Universal Microplate reader (Bio-Tek Instruments).

### 4.8. Statistical Analysis

All results were expressed and plotted as mean ± standard deviation (SD). Data analysis was performed by one-way analysis of variance (ANOVA). Statistical significance was considered for *p*-values of <0.05. Statistical analysis was only performed wherever more information from the results were needed.

## 5. Conclusions

Electrospun fiber mats were prepared by mixing different volumes of gelatin, collagen, and PEG solutions with and without complexes generated from non-viral delivery agents. SEM images revealed that high volume of collagen in gelatinl-PEG mats affected the quality and the structure of the electrospun fibers. The incorporation of the complexes into different Gelatin-Collagen-PEG mats showed the negative effect of collagen on the transfection efficiency of gWIZ-GFP complexes and ALP activity induction activity of pBMP-2 complexes as compared to free complexes. All results showed a consistently negative effect of high collagen concentration on the bioactivity of complexes when the mats were fabricated from a mixture of complexes and collagen material. To overcome this limitation, we have fabricated double-layered scaffolds that contained a collagen mat as a first layer and Gel-Col-PEG/complexes as a second layer, which prevented the contact of collagen with the complexes during the electrospinning. Such scaffolds were successfully able to increase the ALP activity in C2C12 cells compared at a level equivalent to the mono-layer mats without collagen. These multilayered scaffolds can be applied for bone regeneration and other gene delivery applications and future preclinical studies are planned to explore the regenerative activity of such electrospun gene-activated mats.

## Figures and Tables

**Table 1:** Average fiber diameter and bead size of different volume ratio Gelatin-Collagen-PEG electrospun mats without and with pDNA complexes.

Samples	Average Diameter (nm)			
	Without Polyplexes		With Polyplexes	
	Fiber	Beat	Fiber	Beat
**Gel-Col-PEG (100-0-100)**	90 ± 20	663 ± 215	97 ± 5.8	770 ± 111
**Gel-Col-PEG (75-25-100)**	140 ± 10	706 ± 95	126 ± 32.1	330 ± 27
**Gel-Col-PEG (50-50-100)**	103 ± 6	867 ± 343	87 ± 21	1080 ± 329
**Gel-Col-PEG (0-100-0)**	146 ± 38	-	177 ± 31	770 ± 99

**Table 2:** Size and ζ-potential of original free particles and particles released from different Gelatin-Collagen-PEG mats. Only All-Fect/pDNA/pASP complexes were entrapped in mats. ND: Not detected.

**Free Complexes**	**Particle Size (nm)**		**ζ-Potential (mV)**	
ALL-Fect/pDNA	101.3 ± 2.0		38.0 ± 2.6	
ALL-Fect/pDNA/pASP	90.0 ± 1.2		35.5 ± 0.5	
**Complexes Released from Mats**	**no** **complexes**	**with** **complexes**	**no** **complexes**	**with** **complexes**
Gel-Col-PEG (100-0-100)	ND	113.3 ± 0.9	ND	16.1 ± 1.4
Gel-Col-PEG (75-25-100)	ND	85.8 ± 2.3	ND	16.8 ± 0.5
Gel-Col-PEG (50-50-100)	ND	45.7 ± 1.5	ND	11.9 ± 5.0
Gel-Col-PEG (0-100-0)	ND	42.0 ± 3.2	ND	10.5 ± 2.7

## Data Availability

Data is contained within the article and supplementary material.

## Supplementary Materials

The following are available online at https://www.mdpi.com/article/10.3390/ph14070666/s1, Figure S1: Correlation function and size distribution of particles released from different volume ratio Gel-Col-PEG mats (A-D) in comparison to mats without particles., Figure S2: The complexes were incubated in trypsin for only 5 min to dissolve the mats prior to any further testing. For assessment here, up to 30 min incubation with trypsin is used to ensure full exposure of the complexes.
