# Supplementary material for: Delivery of Bioactive Gene Particles via Gelatin-Collagen-PEG-Based Electrospun Matrices
Source: Pharmaceuticals (Basel). 2021 Jul 12;14(7):666. doi: 10.3390/ph14070666 (PMC8308744; doi:10.3390/ph14070666)
Supplement: Supplementary file 1 [file pharmaceuticals-14-00666-s001.zip › pharmaceuticals-1254494-supplementary.pdf]

Supplementary Information

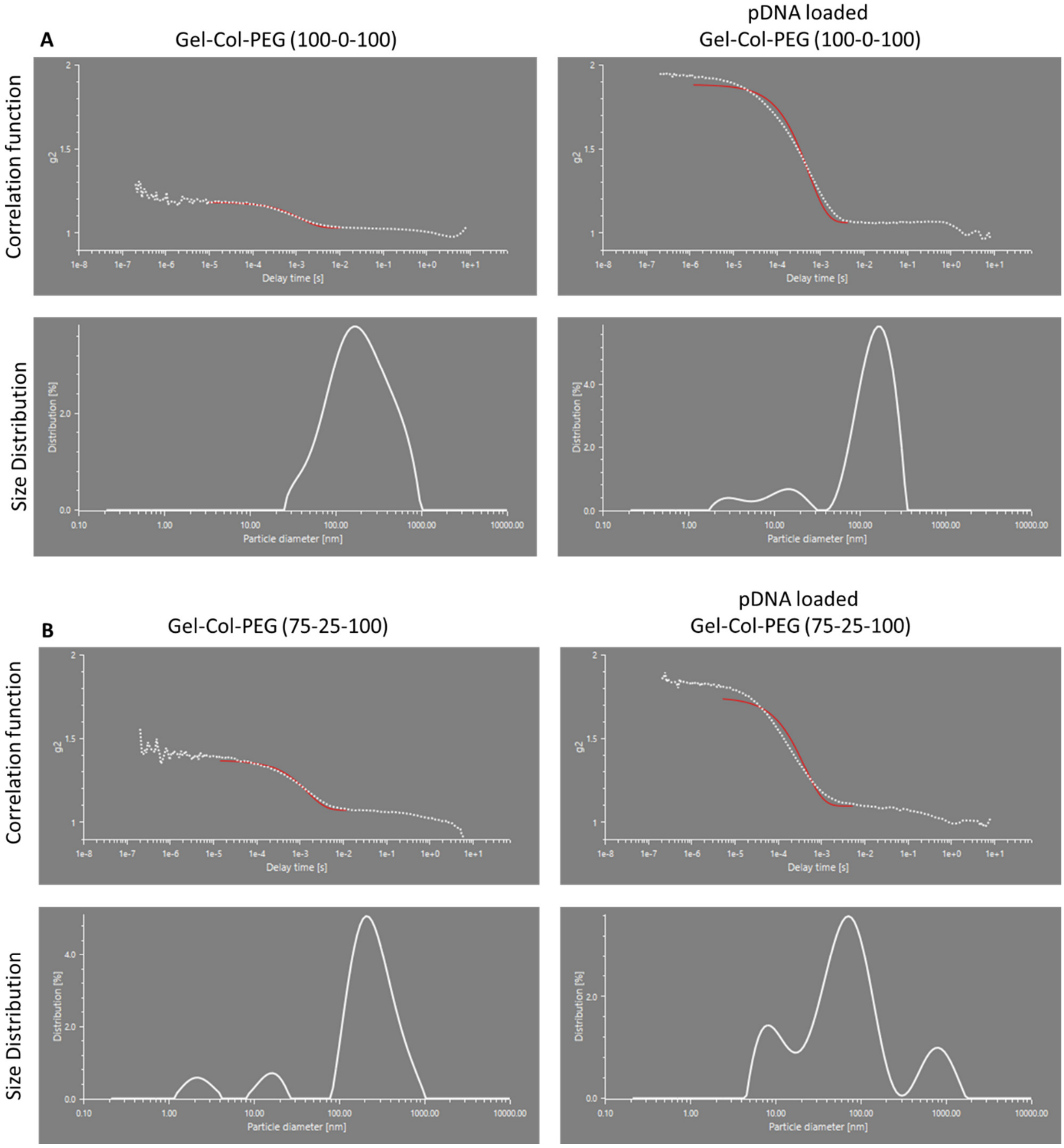

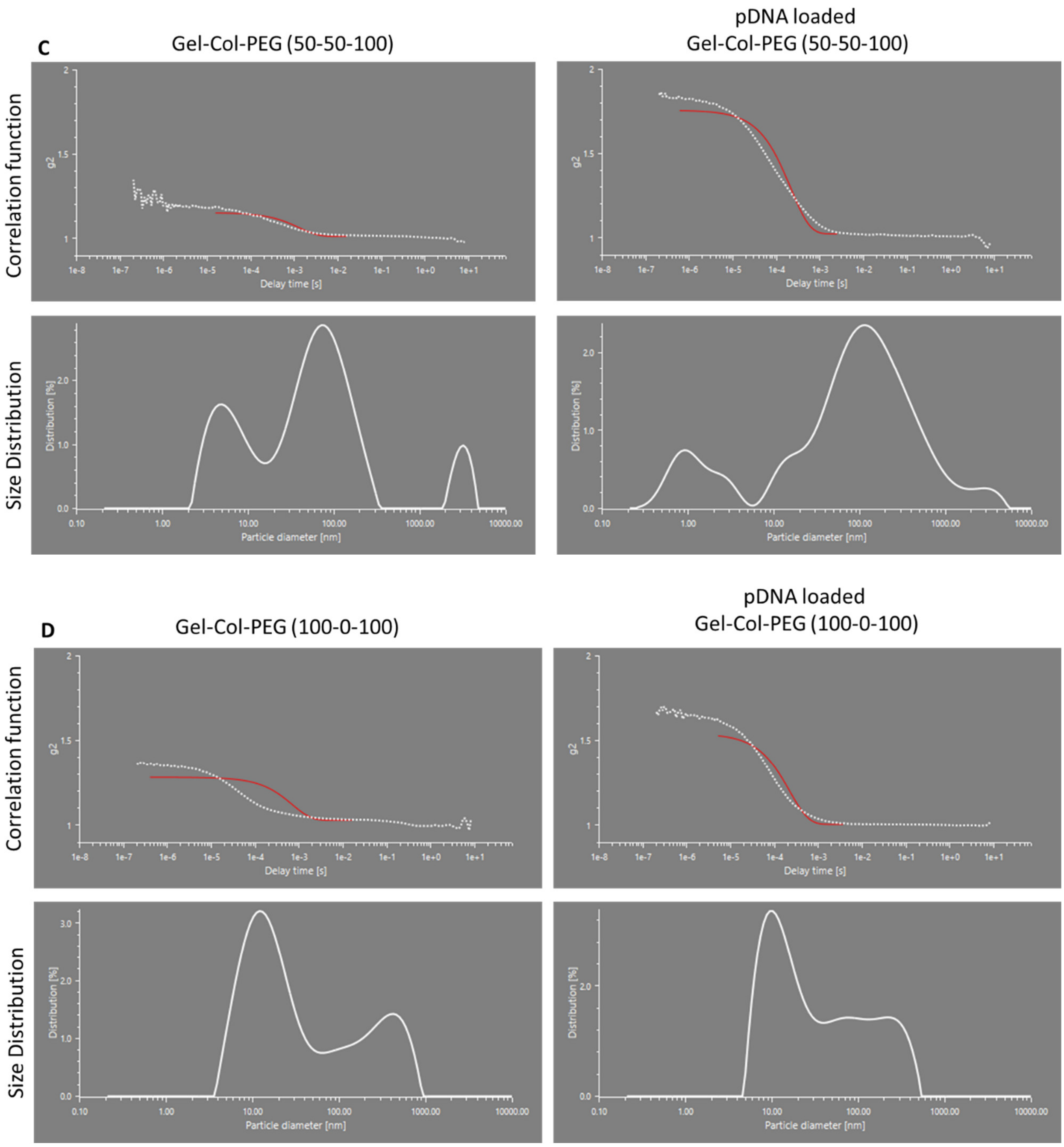

Figure S1. Correlation function and size distribution of particles released from different volume ratio Gel-Col-PEG mats (A-D) in comparison to mats without particles.

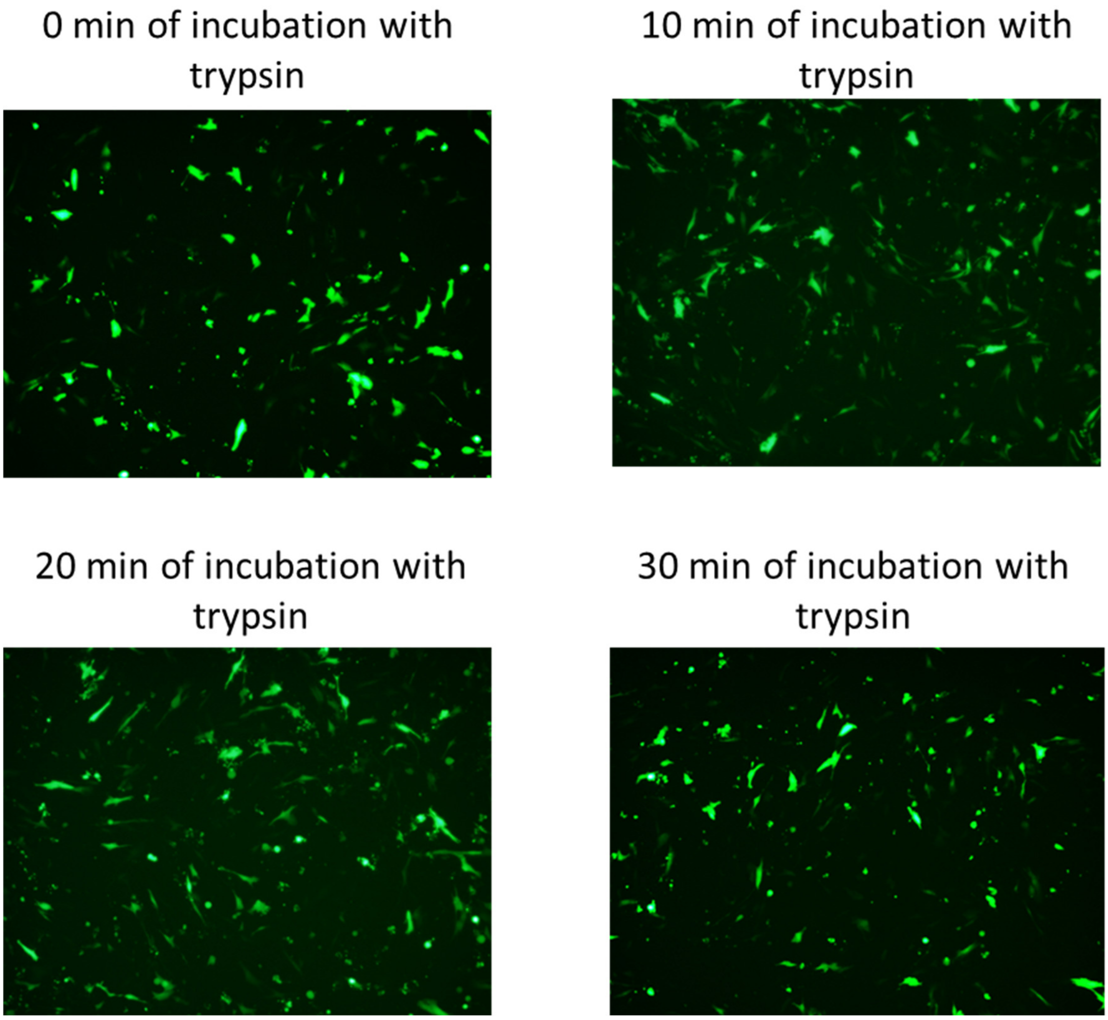

Figure S2. The complexes were incubated in trypsin for only 5 min to dissolve the mats prior to any further testing. For assessment here, up to 30 min incubation with trypsin is used to ensure full exposure of the complexes.
